# Molecular characterization of cytidine monophospho-N-acetylneuraminic acid hydroxylase (*CMAH*) associated with the erythrocyte antigens in dogs

**DOI:** 10.1186/s40575-019-0076-1

**Published:** 2019-11-07

**Authors:** Yumiko Uno, Shota Kawakami, Kazuhiko Ochiai, Toshinori Omi

**Affiliations:** Department of Basic Science, Nippon Veterinary and Life Science University, 1-7-1 Kyonan-cho, Musashino-shi, Tokyo, 180-8602 Japan

**Keywords:** Cytidine monophospho-N-acetylneuraminic acid hydroxylase, Dog, N-glycolylneuraminic acid, N-acetylneuraminic acid, Mutation

## Abstract

**Background:**

N-glycolylneuraminic acid (Neu5Gc) is synthesized from its precursor N-acetylneuraminic acid (Neu5Ac) by cytidine-5′-monophospho-N acetylneuraminic acid hydroxylase (CMAH), which is encoded by the *CMAH* gene. Most mammals have both Neu5Gc and Neu5Ac, but humans and ferrets have only Neu5Ac because of loss-of-function mutations. Dogs and cats are polymorphic for Neu5Gc and Neu5Ac expression like cats, in which the *CMAH* gene is responsible for the AB Blood group system. Although the *CMAH* gene has been characterized in many species, not much is known about it in dogs. In this study, we cloned the dog *CMAH* cDNA, and performed mRNA expression analysis of this gene in several organs. We also identified single nucleotide polymorphisms (SNPs) in the *CMAH* gene.

**Results:**

We cloned the 1737-bp open reading frame of the dog *CMAH* gene. This gene consists of at least 14 coding exons and codes for a polypeptide of 578 amino acids and is located on chromosome 35. The amino acid identities of dog *CMAH* with the corresponding sequences from cat, pig, chimpanzee, mouse, and rat were high (89 to 93%). RT-PCR analysis showed that the dog *CMAH* cDNA was expressed in various tissues. We identified four exonic SNPs (three synonymous and one non-synonymous), 11 intronic SNPs, and an indel in 11 dog breeds by analyzing the nucleotide sequences of the 14 exons, including the coding region of *CMAH.* In the genotype of the non-synonymous SNP, c.554 A > G (p.Lys185Arg), in a total of 285 dogs of seven different breeds, the allele G was widely distributed, and the allele A was the most frequent in the Shiba dogs. The dogs expressing Neu5Ac did not carry the loss-of-function deletion of *CMAH* found in humans and ferrets, and it remains unclear whether the point mutations influence the expression of Neu5Ac.

**Conclusions:**

We characterized the canine *CMAH* gene at the molecular level for the first time. The results obtained in this study provide essential information that will help in understanding the molecular roles of the *CMAH* gene in canine erythrocyte antigens.

## Plain English summary

The sialic acids that are commonly found in mammalian cells are N-glycolylneuraminic acid (Neu5Gc) and N-acetylneuraminic acid (Neu5Ac). The enzyme cytidine monophosphate-N-acetylneuraminic acid hydroxylase (CMAH), which is encoded by the *CMAH* gene, catalyzes the conversion of Neu5Ac to Neu5Gc. In humans and ferrets, the CMAH enzyme is inactivated because of a genetic error in the *CMAH* gene, and thus, Neu5Gc is not produced. This represents one of the few differences between man and apes at the protein level, with various potential evolutionary roles, such as in the selection against pathogens and inflammation. In cats, the AB blood groups are a result of mutations in the *CMAH* gene that affect the production of Neu5Gc. Cats with Neu5Gc represent blood type A antigen and cats with Neu5Ac represent blood type B antigen. Dogs can also be categorized on the basis of the presence of Neu5Ac and Neu5Gc. In general, most European dogs have Neu5Ac whereas dogs of East Asian origin may have either. In addition, Neu5Gc is suggested to be the target receptor for pathogens, such as canine parvoviruses and equine influenza A virus in dogs. Although the *CMAH* gene has been well characterized in cats, not much is known about it in dogs. We have characterized the dog *CMAH* gene for the first time and show that it is located on chromosome 35, with a 1737-bp open reading frame, consisting of 14 coding exons which code for a polypeptide of 578 amino acids, Dog *CMAH* cDNA was expressed in various tissues as assessed by RT-PCR. We also identified four exonic and 11 intronic SNPs, and an indel in *CMAH* in 11 dog breeds. One SNP, c.554 A > G (p.Lys185Arg), was found to be widely distributed in 285 dogs from seven breeds. Moreover, the Shiba dog was identified to be the most polymorphic at this locus among the breeds used in the study. We demonstrate, for the first time, the molecular characterization of the canine *CMAH* gene. The results obtained in this study provide essential information that will help in understanding the molecular roles of the *CMAH* gene in the canine erythrocyte antigens.

## Background

Sialic acids are components of carbohydrate chains of glycoconjugates. The sialic acids that are commonly found in mammalian cells are N-glycolylneuraminic acid (Neu5Gc) and N-acetylneuraminic acid (Neu5Ac). The enzyme cytidine monophosphate-N-acetylneuraminic acid hydroxylase (CMAH), which is encoded by the *CMAH* gene, catalyzes the conversion of Neu5Ac to Neu5Gc [1, 2]. Owing to the presence of a non-functional deletion mutant of *CMAH*, humans have only Neu5Ac [3–6]. The absence of Neu5Gc as a consequence of the inactivation of *CMAH* might have affected several aspects of evolutionary and human biology (such as selection against pathogens, neural functions, and inflammation) in multiple ways [7–11]. In xenotransplantation, Neu5Gc is a known xenoantigen of animals for humans [12, 13]. In addition, Neu5Gc is also a factor responsible for red meat syndrome [14], which occurs through the diet [8]. To investigate the role of CMAH, *CMAH* knockout animals, including pigs, cattle and mice, have been produced [13, 15–19].

Several dog and cat breeds are polymorphic for Neu5Gc and Neu5Ac expression. Neu5Gc and Neu5Ac are antigens known to be involved in the feline AB blood group system [20, 21], and are the most significant antigens in transfusion medicine in cats and in neonatal isoerythrolysis [22–26]. The feline AB blood group system consists of A and B antigens and includes blood groups, type A and type B, and the rare blood group type AB. The type A and type B antigens are Neu5Gc and Neu5Ac, respectively. The erythrocytes in the rare blood group, type AB, express both Neu5Gc and Neu5Ac. In cats, the serum contains naturally occurring antibodies against antigens not present in each cat, i.e., 95% of type A cats have antibodies to type B antigen, and 35% of type B cats have antibodies to type A antigen [27]. The Neu5Ac expression results from the non-functional *CMAH* gene as a result of deletion, especially in exon 6. There are several mutations in the *CMAH* gene in cats that could be associated with the loss or reduced activity of the CMAH enzyme (conversion of Neu5Ac to Neu5Gc), controlling the expression of Neu5Ac (B antigen) in type B and type AB cats [28–31].

Dogs can be clearly divided into two groups, namely the Neu5Gc and Neu5Ac groups. The Neu5Gc group has autosomal dominant inheritance of Neu5Ac, as determined by pedigree analysis [32]. A few mongrel dogs, some Kai dogs, Kishu dogs, Japanese spaniel, and Shiba dogs have either Neu5Ac or Neu5Gc. Among these dogs, Shiba are most frequently observed to have Neu5Gc expression on erythrocytes, whereas most European dogs have Neu5Ac expression and no Neu5Gc expression [32, 33].

Polymorphic Neu5Gc and Neu5Ac expression on cat erythrocytes define the blood group systems. The different forms of these sialic acids result in either a new blood type or one of the blood types present in dogs. Löfling et al. recently reported that canine and feline parvoviruses preferentially recognize Neu5Gc [34]. In addition, an equine influenza A (H3N8) virus, with W222 L mutation in hemagglutinin, had increased binding of canine-specific receptors with sialyl Lewis X and Neu5Gc motifs [35].

Although Neu5Gc and Neu5Ac are important molecules, possibly associated with blood groups in dogs and also with infectious diseases, the molecular basis underlying the function of dog *CMAH* is not much understood. Herein, we report, for the first time, the cloning of *CMAH* cDNA, the expression of *CMAH* mRNA in various tissues, and several single nucleotide polymorphisms (SNPs) present in this gene.

## Results

### Cloning of the full-length cDNA of dog *CMAH*

**Fig. 1:**
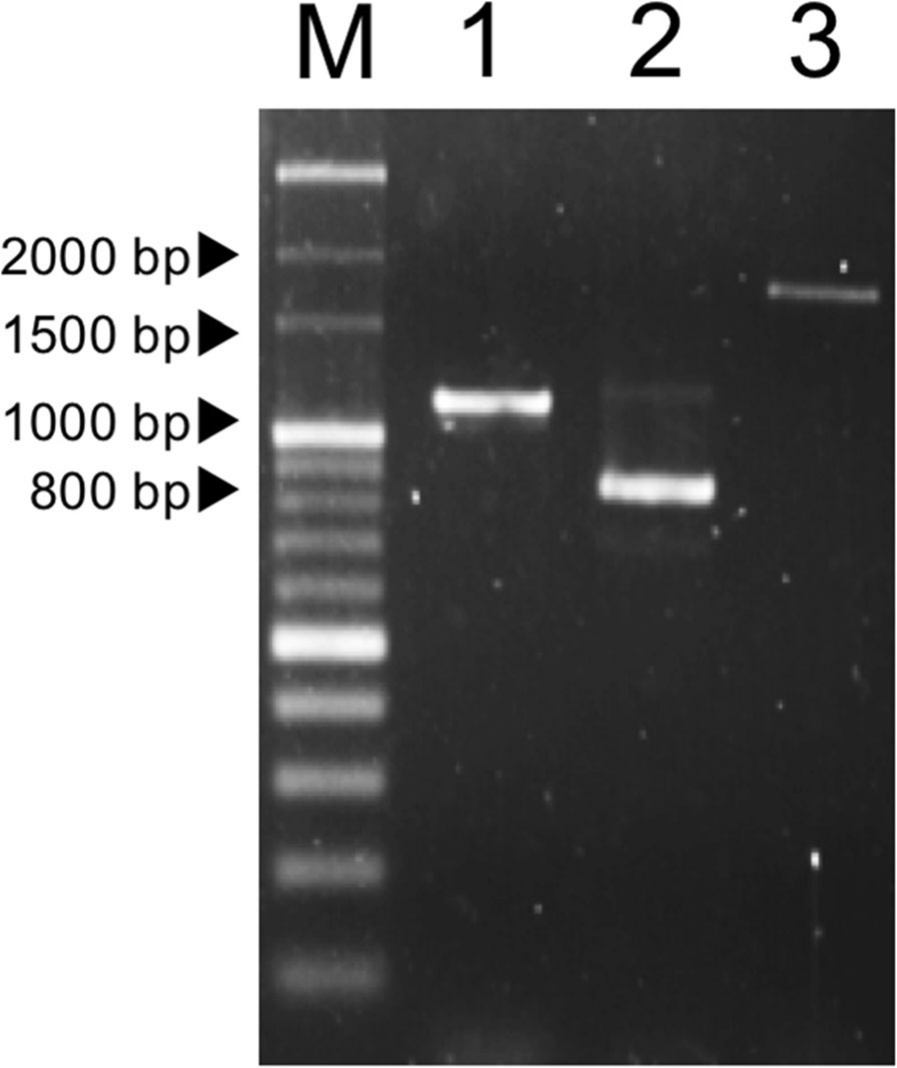
Dog *CMAH* cDNA amplified from total RNA extracted from bone marrow by RT-PCR. M: 100 bp DNA Marker (Bio Regenerations Co. Ltd.), Lane 1: cDNA encompassing exons 1a to 9 (Fragment 1 in Table 3), Lane 2: cDNA encompassing exons 8 to E15 (Fragment 2 in Table 3), 3: Lane cDNA encompassing exons 1a to E15 (Fragment 3 in Table 3)

**Fig. 2:**
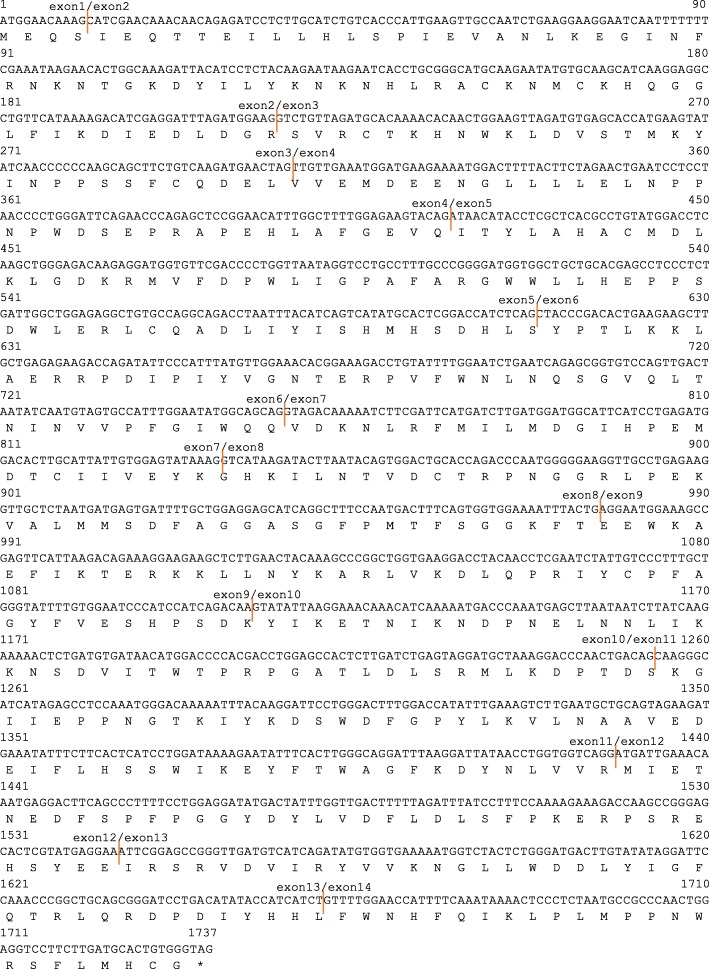
Nucleotide sequence and exon borders of the coding region of the dog *CMAH* gene. Orange bar: exon boundaries resulting from comparison of nucleotide sequence of the dog *CMAH* cDNA with the dog genome sequence (NC_006617.3). Number above each line is number of nucleotide sequences. Under line: Start Codon, *: Stop codon

A 1737-bp open reading frame (ORF) of dog *CMAH* was amplified using cDNA prepared from dog bone marrow by RT-PCR (Fig. 1). The complete nucleotide sequence of the dog *CMAH* coding region was submitted to GenBank (Accession No. AB067771). The cDNA was predicted to encode a protein of 578 amino acid residues (Fig. 2). The ORF of *CMAH* was shown to be composed of 14 exons by comparison with the dog genome sequences (NC_006617.3).

The dog CMAH amino acid sequence was 93% identical with the cat [30], 93% with the chimpanzee, (NP_001009041), 92% with the pig (NP_001106486), 90% with the mouse (NP_001104580), 89% with the rat (NP_001019444.1), and 68% with the zebra fish (NP_001002192) *CMAH* sequences. Multiple alignment of the deduced amino acid sequences of these *CMAH* is shown in Additional file 1. The evolutionary tree generated by the Unweighted Pair Group Method with Arithmetic Mean using Genetyx-MAC is shown in Additional file 2. The human protein (AAC68881) is only 72 amino acids long and is nonfunctional because of a 92-bp frame-shifting exon deletion [3, 4].

### Expression analysis of *CMAH* mRNA in various tissues

**Fig. 3:**
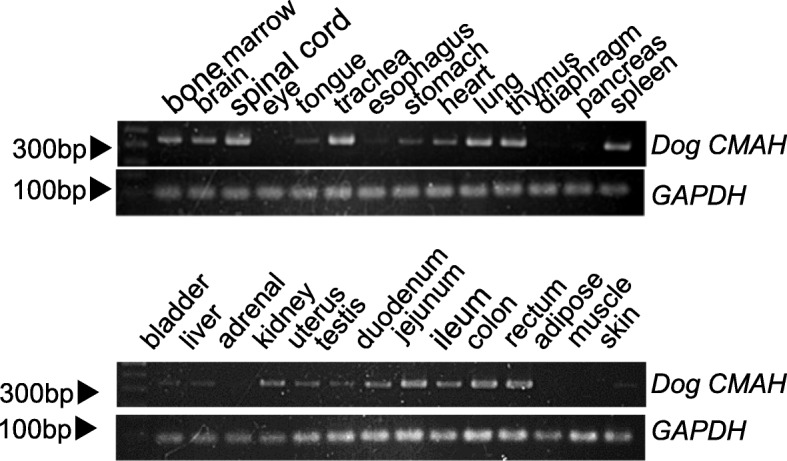
RT-PCR analysis of canine *CMAH* mRNA in various tissues of dog. Dog *CMAH* cDNA encompassing exons 2 to 4 (342 bp) was amplified from various tissues. The integrity of RNA was examined using the primer pair for glyceraldehyde-3-phosphate dehydrogenase (GAPDH)

To determine the expression of canine *CMAH* in different tissues, we performed RT-PCR using total RNA extracted from 28 tissues. The integrity of RNA was examined by amplification of *GAPDH* cDNA. The amplification of dog *CMAH* cDNA, encompassing exons 2 to 4 (342 bp), was observed for bone marrow, brain, spinal cord, tongue, trachea, esophagus, stomach, heart, lung, thymus, spleen, bladder, liver, kidney, uterus, testis, duodenum, jejunum, colon, rectum, and skin samples (Fig. 3). However, no or extremely low amplification was observed in samples from eye, diaphragm, adrenal, adipose, and muscle tissues.

### Discovery of SNPs in the canine *CMAH* gene

**Fig. 4:**
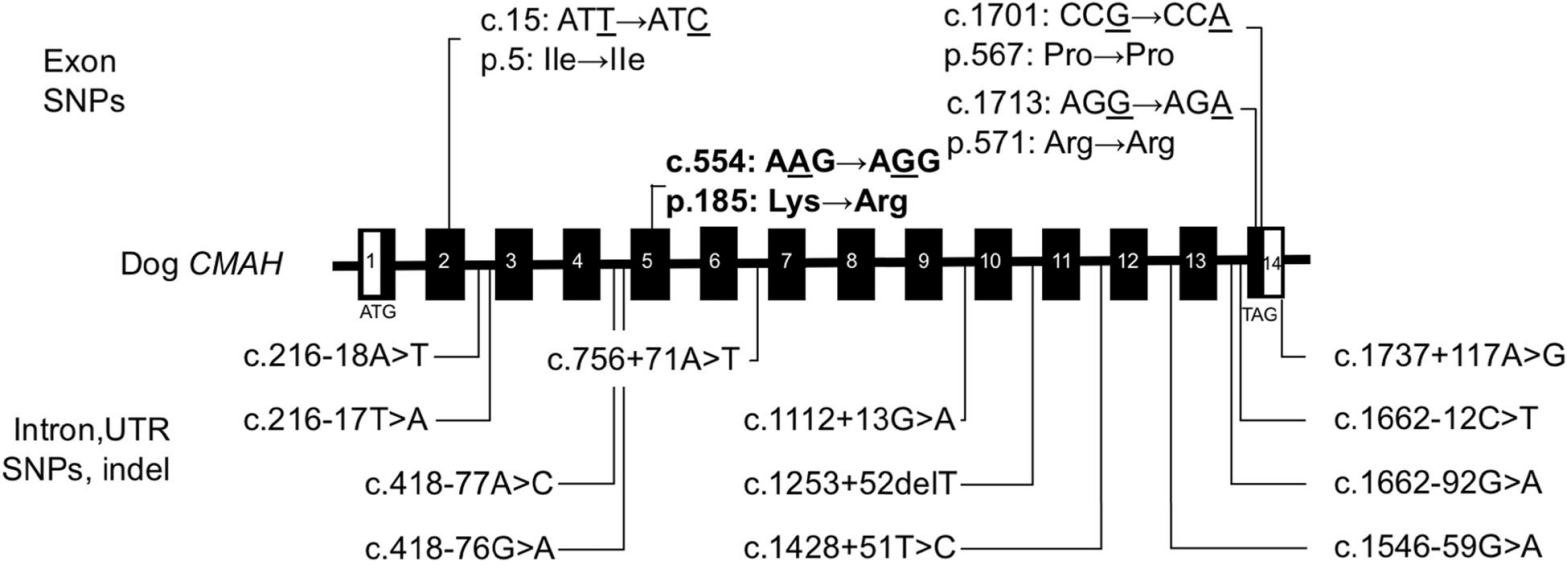
Schematic representation of the DNA polymorphisms detected in canine *CMAH*. Genomic structure of the *CMAH* gene from dog was obtained by comparative analysis of mRNA sequences and whole genome shotgun sequence (Accession No. NC_006617.3, chromosome 35). □: Exon (UTR), ■: CDS, ▬: Intron. The position of the identified DNA polymorphism was numbered from the A of the initiator methionine ATG codon considered to be + 1, as revealed in the exon. In introns, a positive number indicates the number of nucleotides from the previous exon, whereas a negative number indicates the number of nucleotides from the next exon

To identify DNA polymorphisms in the canine *CMAH* gene, 14 regions of the canine gene were individually amplified and sequenced from genomic DNA samples from 11 dogs, each from a different breed. We identified 15 SNPs (4 exonic and 11 intronic) and indels in the 11 dogs, with some found in a single breed, and others in several breeds. The nucleotides in the dog genome sequence (NC_006617.3) corresponding to the polymorphic alleles were considered as wild type in this study. The different DNA polymorphisms of the canine *CMAH* gene are shown in Fig. 4. In addition, the genotype of the canine *CMAH* gene in 11 dogs is also shown in Additional file 3.

### Distribution of c.554 A > G SNP of *CMAH* in different dog breeds

In general, all European dogs have Neu5Ac but no Neu5Gc, while East Asian dogs have either of these two sialic acids [32, 33]. Three of the four exonic SNPs were synonymous: c.15 T > C (p.lle15lle) in exon 2, and c.1701G > A (p.Pro567Pro) and c.1713G > A (p.Arg571Arg) in exon 14. We focused on the one non-synonymous SNP, cc.554A > G (p.Lys185Arg) in exon 5, and determined its distribution in 229 dogs from seven breeds: Chihuahua, French Bulldog, Golden Retriever, Labrador Retriever, Miniature Dachshund, Shiba Dog, and Toy Poodle (Table 1). Although the allele present in the Boxer dog genome sequence (NC_006617.3) was A at position c.554, this appears to be the minor allele (0.062 to 0.025) in the breeds we tested. We found that allele G was widely distributed (0.717 to 0.938) among six of the seven tested breeds. However, the genotyping results for the Shiba dog were different from the six other breeds at position c.554: making the Shiba dog the most polymorphic breed (G:0.665, A:0.335) at c.554 A > G.

**Table 1:** Distribution of c.554 A > G SNP of *CMAH* in different dog breeds

	Chihuahua (*n* = 34)	French Bulldog (*n* = 35)	Golden Retriever (*n* = 30)	Labrador Retriever (*n* = 32)	Miniature Dachshund (*n* = 33)	Shiba Dog (*n* = 31)	Toy Poodle (*n* = 34)
	Number (%)	Number (%)	Number (%)	Number (%)	Number (%)	Number (%)	Number (%)
Genotype frequency							
AA	3 (8.8)	0 (0)	2 (6.7)	0 (0)	1 (3.0)	4 (12.9)	0 (0)
AG	11 (32.3)	4 (11.4)	13 (43.3)	4 (12.5)	7 (21.2)	14 (45.2)	6 (17.6)
GG	20 (58.9)	31 (88.6)	15 (50.0)	28 (87.5)	25 (75.8)	13 (41.9)	28 (82.4)
Allele frequency							
A	0.250	0.057	0.283	0.062	0.136	0.355	0.088
G	0.750	0.943	0.717	0.938	0.864	0.645	0.912

*n* number of sample

The Argenine at codon 185 of the CMAH protein (allele G at position c.554) is conserved in the various species listed in Additional file 1 which include the dingo (*Canis lupus dingo)* (NCBI Reference Sequence: XM_025442865.1). Thus, the allele G may represent the wild type for position c.554.

### Correlation of the *CMAH* gene at the c.554A > G locus in dogs with presence or absence of Neu5Ac expression

To investigate the *CMAH* polymorphisms in dogs with the presence or absence of Neu5Ac expression, the positive or negative phenotype for the binding of lectin with Neu5Ac was determined in 56 Shiba dogs and 29 Labrador Retrievers. Thereafter, the Shiba dogs were divided into two groups, positive (78.6%) or negative (21.4%), with regard to the binding of lectin with Neu5Ac. In contrast, all the Labrador Retriever dogs were positive for Neu5Ac (Table 2). We determined the nucleotide sequence of the coding region of the dog *CMAH* gene using genomic DNA extracted from the Shiba dogs that were positive or negative for the binding of lectin with Neu5Ac. Genomic analysis of the dog *CMAH* gene in the coding region showed that there were no loss-of-function deletions related to the Neu5Ac expression in the Shiba dog, unlike humans and ferrets [3, 4, 36].

**Table 2:** Correlation of the *CMAH* gene at the c.554A > G locus in dogs with presence or absence of Neu5Ac expression

	Shiba Dog (*n* = 56)		Labrador Retriever (*n* = 29)		Total (*n* = 85)	
Phenotype	Positive (%)	Negative (%)	Positive (%)	Negative (%)	Positive (%)	Negative (%)
	44 (78.6)	12 (21.4)	29 (100)	0(0)	73 (85.9)	12 (14.1)
Genotype						
AA	10 (22.7)	0 (0)	0(0)	0(0)	10 (13.7)	0 (0)
AG	21 (47.7)	4 (33.3)	3 (10.3)	0(0)	24 (32.9)	4 (33.3)
GG	13 (29.5)	8 (66.7)	26 (89.7)	0(0)	39 (53.4)	8 (66.7)
Allele frequency						
A	0.466	0.167	0.052	0	0.301	0.167
G	0.534	0.833	0.948	0	0.699	0.833

*n* number of sample

Erythrocyte Neu5Ac expression was determined in reaction of lectin for Neu5Ac

Since the presence of several point mutations are associated with Neu5Ac expression in cats [28–30], we investigated whether there was a correlation in dogs with the presence or absence of Neu5Ac expression with the position c.554A > G (Table 2). For the dogs that had positive binding of lectin with Neu5Ac, the genotype frequencies in Shiba dogs were: AA 22.7%, AG 47.7% and GG 29.5%, while in Labradors the genotype frequencies were: AA 0%, AG 10.3% and GG 89.7%. However, in Shiba dogs that were negative for the binding, the genotype frequencies were AA 0%, AG 33.3% and GG 66.7%. These results do not clarify whether the point mutations at c.554A > G influence the expression of Neu5Ac.

## Discussion

Although the *CMAH* gene has been characterized in many species, not much is known about this gene in dogs. We cloned the dog *CMAH* cDNA, and performed mRNA expression analysis of this gene in several different tissues, and identified SNPs in the *CMAH* gene.

The molecular characterization of canine *CMAH*, which is responsible for the synthesis of Neu5Gc from Neu5Ac, involved cloning the cDNA, assessing the mRNA expression in several different tissues, identifying the SNPs present in the gene, and examining the distribution of one of the identified SNPs in different dog breeds.

The dog *CMAH* gene consists of at least 14 coding exons and encodes a protein of 578 amino acids. It is located on chromosome 35. The amino acid sequence was found to be highly similar (89–93%) with the corresponding sequences in cat, pig, chimpanzee, mouse, and rat. Interestingly, the amino acid sequence of *CMAH* from *Canis lupus familiaris* (dog) showed 100% identity with the predicted sequence from *CMAH* mRNA from *Canis lupus dingo* (dingo) in the NCBI database (Accession No.: XM_025442865).

In cats, *CMAH* is expressed in most of the tissues (Additional file 4). In dogs, this gene was observed to be expressed in many tissues; but it was not expressed in all of the tissues. The breed or phenotype (Neu5Gc or Neu5Ac) of the dog from which the commercial cDNA sample was prepared was unknown.

We demonstrate the presence of the *CMAH* gene and its expression in dogs, which suggests that dogs also have the CMAH enzyme, like other mammals. Neu5Ac is a precursor of other diverse sialic acids, including Neu5Gc. Expression of Neu5Ac by elimination of Neu5Gc, results from two different genetic mechanisms: the loss-of-function deletion (as in humans [3, 4] and ferrets [36]), or point mutation (as in cats [28–31]). It is known that all European dogs have Neu5Ac and they do not have Neu5Gc; however, Shiba dogs can have either of the two [32]. In this study, no deletion in the coding region of *CMAH* was found in Labrador Retrievers or Shiba dogs expressing Neu5Ac, (Tables 1 and 2, Fig. 4). These results suggest that the genetic mechanism of expression of Neu5Ac is not a loss-of-function deletion in the coding region of the *CMAH* gene but a point mutation.

Several non-synonymous SNPs were reported in the Neu5Ac expressing cats (Types B and AB) [28–31]. In canine *CMAH*, we detected a non-synonymous, c.554A > G (p.Lys185Arg), mutation in exon 5. The allele G or GG genotypes at c.554 A > G in the dog *CMAH* gene were widely distributed in seven breeds of dog (Table 1). Since most European dogs express Neu5Ac, but not Neu5Gc [32, 33], we assumed that the allele G at c.554 A > G was associated with Neu5Ac. This hypothesis matched the genotype results from 29 Labrador Retriever with Neu5Ac expression, but this was not the case for Shiba Dogs positive for the binding of lectin with Neu5Ac (Table 2). Thus, it remains unclear whether the amino acid substitution (p.Lys185Arg) caused by the c.554A > G SNP influences the Neu5Ac expression based on the CMAH activity. Recently, a promoter region responsible for the intestine-specific regulation of porcine *CMAH* was found [37, 38]. Future studies will need to investigate the promoter region of dog *CMAH* to determine the regulation of the expression of this gene.

Neu5Gc is considered to be a target receptor for pathogens, such as canine parvoviruses and equine influenza A (H3N8) virus in dogs [34, 35]. In humans, Neu5Gc has been suggested to be a target receptor for pathogens as well as a tumour marker and a major xenoantigen, and is also reported to be involved in inflammation [7–11]. In addition, the antigens of Neu5Gc and Neu5Ac expressed on dog erythrocyte membrane may, or may not, help define the dog blood group system. The characterization of dog *CMAH* gene would help understand the roles of Neu5Ac and Neu5Gc in dog biology.

## Conclusion

We identified a 1737-bp ORF of the canine *CMAH* gene. This gene consists of at least 14 exons, encoding a 578-amino acid protein, and is located on chromosome 35. The RT-PCR analysis showed that the dog *CMAH* cDNA was expressed in several tissues. There were no loss-of-function deletion mutants of *CMAH* in dogs expressing Neu5Ac. We identified a non-synonymous c.554A > G (p.Lys185Arg) SNP in exon 5. The Shiba dog was most polymorphic (G: 0.665, A: 0.335) at c.554 A > G, in contrast to the other six breeds investigated. Whether this SNP influences the expression of Neu5Ac remains unknown. The results of the present study provide useful information for understanding the molecular roles of the *CMAH* gene in the canine erythrocyte antigens.

## Methods

### Dogs

Genomic DNA samples extracted from the blood of one dog each from 11 different breeds (Miniature Dachshund, Welsh Corgi, Labrador Retriever, Shetland Sheepdog, Beagle, Yorkshire Terrier, Dobermann, Whippet, Weimaraner, Papillon, and Shiba dog) were used for detection of mutations in *CMAH*. For genotyping, genomic DNA from 197 dogs belonging to seven different breeds (34 Chihuahua, 35 French Bulldog, 30 Golden Retriever, 32 Labrador Retriever, 33 Miniature Dachshund, 31 Shiba Dog, and 34 Toy Poodle) was used. Blood samples from 56 Shiba dogs and 29 Labrador Retriever were used for association study between the Neu5Ac expression and the genotype. In each case, genomic DNA was extracted from whole blood using the Puregene kit (Qiagen, Valencia, CA, USA), according to the manufacturer’s instructions.

The blood samples of random dog populations were provided by the Department of Veterinary Clinical Pathology, Nippon Veterinary and Life Science (NVLU). The samples were collected at the Veterinary Medical Teaching Hospital at NVLU, with the written consent of dog owners. Sample collection was only handled by licensed veterinarians. This study was approved by the Experimental Animal Ethics Committee at NVLU.

### Cloning of *CMAH* cDNA

We amplified three different fragments using cDNA prepared from the bone marrow, with three primer pairs (dCMAH-ElaF/dCMAH-E9R, dCMAH-E8F/dCMAH-E15R, and dCMAH-ElaF/dCMAH-E15R), including the ORF for canine *CMAH*. These primers were designed based on the draft sequence of the canine genome (GenBank Accession No. NC_006617.3) and our previous study [30]. RT-PCR was performed using FastStart *Taq* DNA polymerase (Roche Diagnostics, Mannheim, Germany), according to the manufacturer’s instructions. The 25-μL RT-PCR mixture contained 0.2 μL cDNA, 2.5 μL 10X PCR buffer with 20 mM MgCl_2_, 0.5 μL 10 mM dNTP, 1 μL 20 pmol upstream PCR primer, 1 μL 20 pmol downstream PCR primer, and 2 U FastStart *Taq* DNA polymerase. PCR amplification was performed using the following temperature profile: 94 °C for 2 min, followed by 35 cycles at 94 °C for 1 min, 55 °C for 1 min, 72 °C for 3 min, and a final extension at 72 °C for 7 min. The list of primers and PCR conditions used in this study are presented in Table 3. The PCR products were electrophoresed on a 2% agarose gel with DNA markers as size standards and visualized by ethidium bromide staining. The PCR products, purified using High Pure PCR Product Purification Kit (Roche, Schweiz), were Sanger sequenced [39] and analyzed with a 3730 Genetic Analyzer (Applied Biosystems).

**Table 3:** List of primer used for analysis of the canine *CMAH* gene

Amplicon	Primer name	Primer sequences (5′-3′)	Location*	Annealing (°C/s)	Predicted product size (bp)	Genbank Accession No., [Ref]
cDNA cloning						
Fragment 1	dCMAH-cE1aF	CTGTTTTGTGCAGTTTGGCCTCTT	exon 1 (5′-UTR)	*55/60	1146	[30]
	dCMAH-cE9R	TTGTCTGATGGATGGGATTCCACA	exon 9			NC_006617.3
Fragment 2	dCMAH-cE8F	CCTGAGAAGGTTGCTCTAATGA	exon 8	*55/60	925	NC_006617.3
	cDMAH-cE15R	TGATCAAGATGTAGCGTCAGTAAAT	exon 15 (3′-UTR)			NC_006617.3
Fragment 3	dCMAH-cE1aF	CTGTTTTGTGCAGTTTGGCCTCTT	exon 1 (5′-UTR)	*55/180	1852	[30]
	cDMAH-cE15R	TGATCAAGATGTAGCGTCAGTAAAT	exon 15 (3′-UTR)			NC_006617.3
SNP discovery						
Fragment 1	dCMAH-gE1F	CTCCAGGCTGCCGTCCTTCTA	Promoter	*55/30	227	NC_006617.3
	dCMAH-gE1R	CAACAGCTTTCAGTTCTTGAAT	intron 1			NC_006617.3
Fragment 2	dCMAH-gE2F	GCCTGGATACTTGGAGGGAGG	intron 1	*55/30	393	NC_006617.3
	dCMAH-gE2R	TGATCAGAGAGATGTCCTAAGT	intron 2			NC_006617.3
Fragment 3	dCMAH-gE3F	AGTATTATCTCCTAATGGTTT	intron 2	*55/30	369	NC_006617.3
	dCMAH-gE3R	AAGTCCGACTCTACACAGTTT	intron 3			NC_006617.3
Fragment 4	dCMAH-gE4F	TGAGTTGGTGTTGGTCTTAAG	intron 3	*55/30	483	NC_006617.3
	dCMAH-gE4R	TCCTAAGAAATTTGCTTAATAG	intron 4			NC_006617.3
Fragment	dCMAH-gE5F	TTGAGCATTCTTAGAAGCGAA	intron 4	*55/30	500	NC_006617.3
	dCMAH-gE5R	CCTCACTCTGAGTGGTATATA	intron 5			NC_006617.3
Fragment 6	dCMAH-gE6F	AATTCCTTGCTTCTTGATCAACA	intron 5	*55/30	334	NC_006617.3
	dCMAH-gE6R	ATCTTAAAGTAACTACCTCATCTAC	intron 6			NC_006617.3
Fragment 7	dCMAH-gE7F	GAAGGATTTCTTTCCAGATGAGC	intron 6	*55/30	254	NC_006617.3
	dCMAH-gE7R	CAGATGGCTGTTATCACCCTT	intron 7			NC_006617.3
Fragment 8	dCMAH-gE8F	GGACATAAGTGATGCTTCTCTA	intron 7	*55/30	445	NC_006617.3
	dCMAH-gE8R	GGCGCTAAACCACTAAGCCAC	intron 8			NC_006617.3
Fragment 9	dCMAH-gE9F	TTCCTGTGTTAGTCTATCCAT	intron 8	*55/30	386	NC_006617.3
	dCMAH-gE9R	AAGCTCCGTGCTCTCAGGCAG	intron 9			NC_006617.3
Fragment 10	dCMAH-gE10F	TAAGTGGATAGGATTGTGAAG	intron 9	*55/30	373	NC_006617.3
	dCMAH-gE10R	CCTACAAATAAGGACTTGTCA	intron 10			NC_006617.3
Fragment 11	dCMAH-gE11F	GATAAGAGAACTTTCCTGTAT	intron 10	*55/30	433	NC_006617.3
	dCMAH-gE11R	TCCGTGAAGCAGTTGGTAGCG	intron 11			NC_006617.3
Fragment 12	dCMAH-gE12F	CATTGCTATCAATTAAGGCTG	intron 11	*55/30	360	NC_006617.3
	dCMAH-gE12R	GTAAAGTAAGGTGTTAACAAT	intron 12			NC_006617.3
Fragment 13	dCMAH-gE13F	AGCCTGTCATATCTACTCCAT	intron 12	*55/30	393	NC_006617.3
	dCMAH-gE13R	GAAATGAAGCCCTAATGGTGG	intron 13			NC_006617.3
Fragment 14	dCMAH-gE14F	CAGTATGGAAGCACCATCTCT	intron 13	*55/30	353	NC_006617.3
	dCMAH-gE14R	ATGTTCTTGCAATGTTAGCTC	intron 14			NC_006617.3
mRNA expression						
CMAH	dCMAH-cE2F	TGTGCAAGCATCAAGGAGGC	exon 2	*55/30	342	NC_006617.3
	dCMAH-cE4R	AGGCAGGACCTATTAACCAG	exon 4			NC_006617.3
GAPDH	dGAPDH-cE1/2	CACAGTCAAGGCTGAGAACG	exon 1/2	*55/30	101	[37]
	dGAPDH-cE2	CACCAGCATCACCCCATTT	exon 2			[37]

Number of exon is detemined by comaparison between Ac. No. LC382414 and NC_006617.3 in this study

NC_006617.3: *Canis lupus familiaris* breed boxer chromosome 35, CanFam3.1, whole genome shotgun sequence

*d* dog, E: exon, *F* Forward primer, *R* reverse primer, *number* exon number, *c* designed, *g* designed from genomic sequence in primer name

### Expression of *CMAH* in canine tissues

The expression analysis of *CMAH* was done with RT-PCR using cDNA from various canine tissues provided by Zyagen (San Diego, CA, USA) and Biochain Institute Inc. (Newark, CA, USA). We used cDNAs from bone marrow, brain, spinal cord, eye, tongue, trachea, esophagus, stomach, heart, lung, thymus, thymus, diaphragm, pancreas, spleen, bladder, liver, adrenal, kidney, uterus, testis, duodenum, jejunum, ileum, colon, rectum, adipose, muscles, and skin. For each sample, the *CMAH* cDNA was amplified from exons 2 to 4. The canine *GAPDH* gene was used as an internal control [40]. The conditions and primers used for RT-PCR are shown in Table 3. The PCR products were electrophoresed on a 2% agarose gel with DNA markers as size standards and visualized by ethidium bromide staining.

### Detection of *CMAH* mutations

Fourteen exons (exons 1a to 14) of *CMAH* containing coding regions were amplified from genomic DNA samples prepared from one dog each from 11 breeds by PCR (Table 3), and their sequences were determined. The DNA polymorphisms were identified by comparing each sequence with the reference sequence (Canine Genome Draft, NC_006617.3) using the BLAST tool available at the National Center for Biotechnology Information website and GENETYX program Ver. 11 (GENETYX Corporation, Tokyo, Japan). The nucleotides in the reference sequence at the mutated position were regarded as the wild type. The positions of the identified DNA polymorphisms were numbered considering A of the initiator methionine ATG codon as + 1 in the case of exon. In the case of introns, a positive number indicates the number of nucleotides from the previous exon, whereas a negative number indicates the number of nucleotides from the next exon.

### C.554A > G SNP genotyping

To characterize a missense SNP (c.554A > G SNP) in canine *CMAH*, identified in this study, we investigated its distribution by analysis of the sequences of the *CMAH* exons in seven different breeds using 197 genomic DNA samples.

We also determined the association between the c.554A > G SNP genotype and Neu5Ac expression. A total of 56 whole blood samples from Shiba dogs were used for this experiment. The lectin (wheat germ agglutinin) solution for Neu5Ac was prepared using the elution of the card recognizing the B (Neu5Ac) antigen in the RapidVet®-H Feline blood typing kit (Kyoritsu Seiyaku Corporation, Tokyo, Japan). A 3% suspension of RBCs from 56 Shiba dogs was diluted with physiological saline and mixed with the lectin solution recognizing Neu5Ac in 12 × 75 mm tubes, at room temperature, and centrifuged at 3000 rpm for 15 s. Agglutination was considered positive if RBCs remained agglutinated after the tubes were gently shaken. Thereafter, we performed the c.554A > G SNP genotyping using genomic DNA extracted from 56 whole blood samples.

## Supplementary information

**Additional file 1 **Multiple alignment of the deduced amino acid sequences of *CMAH* in various species. Dot(.): same sequence for dog CMAH, dash (−): missing sequence for dog CMAH, *: position of amino acid residue at 185 that corresponded to dog CMAH (p.Lys185Arg).

**Additional file 2 **Evolutionary tree generated by Unweighted Pair Group Method with Arithmetic Mean from *CMAH* amino acid sequences using Genetyx-MAC.

**Additional file 3.** List of genotypes of 15 single nucleotide polymorphisms (4 exonic and 11 intronic) and an indel in 11 dogs, each from a different breed. Red character shows the nucleotide mutation revealed in this study.

**Additional file 4 **RT-PCR analysis of feline *CMAH* mRNA expression in different tissues of cat.

## Data Availability

All data generated and analyzed during this study are included in this published article and in the supplementary information files.

## Abbreviations

ACBT: Beta Actin
CMAH: Cytidine-5′-monophospho-N acetylneuraminic acid hydroxylase
GAPDH: Glyceraldehyde-3-phosphate dehydrogenase
Neu5Ac: N-acetylneuraminic acid
Neu5Gc: N-glycolylneuraminic acid
ORF: Open Reading Frame
PCR: Polymerase Chain Reaction
RT-PCR: Reverse Transcription Polymerase Chain Reaction
SNPs: Single Nucleotide Polymorphisms
