# Supplementary material for: Molecular characterization of cytidine monophospho-N-acetylneuraminic acid hydroxylase (CMAH) associated with the erythrocyte antigens in dogs
Source: Canine Genet Epidemiol. 2019 Nov 7;6:9. doi: 10.1186/s40575-019-0076-1 (PMC6842231; doi:10.1186/s40575-019-0076-1)
Supplement: Supplementary file 1 — Additional file 1 Multiple alignment of the deduced amino acid sequences of CMAH in various species. Dot(.): same sequence for dog CMAH, dash (−): missing sequence for dog CMAH, *: position of amino acid residue at 185 that corresponded to dog CMAH (p.Lys185Arg). [file 40575_2019_76_MOESM1_ESM.docx]

Additional file 1. Multiple alignment of the deduced amino acid sequence of *CMAH* in various species.


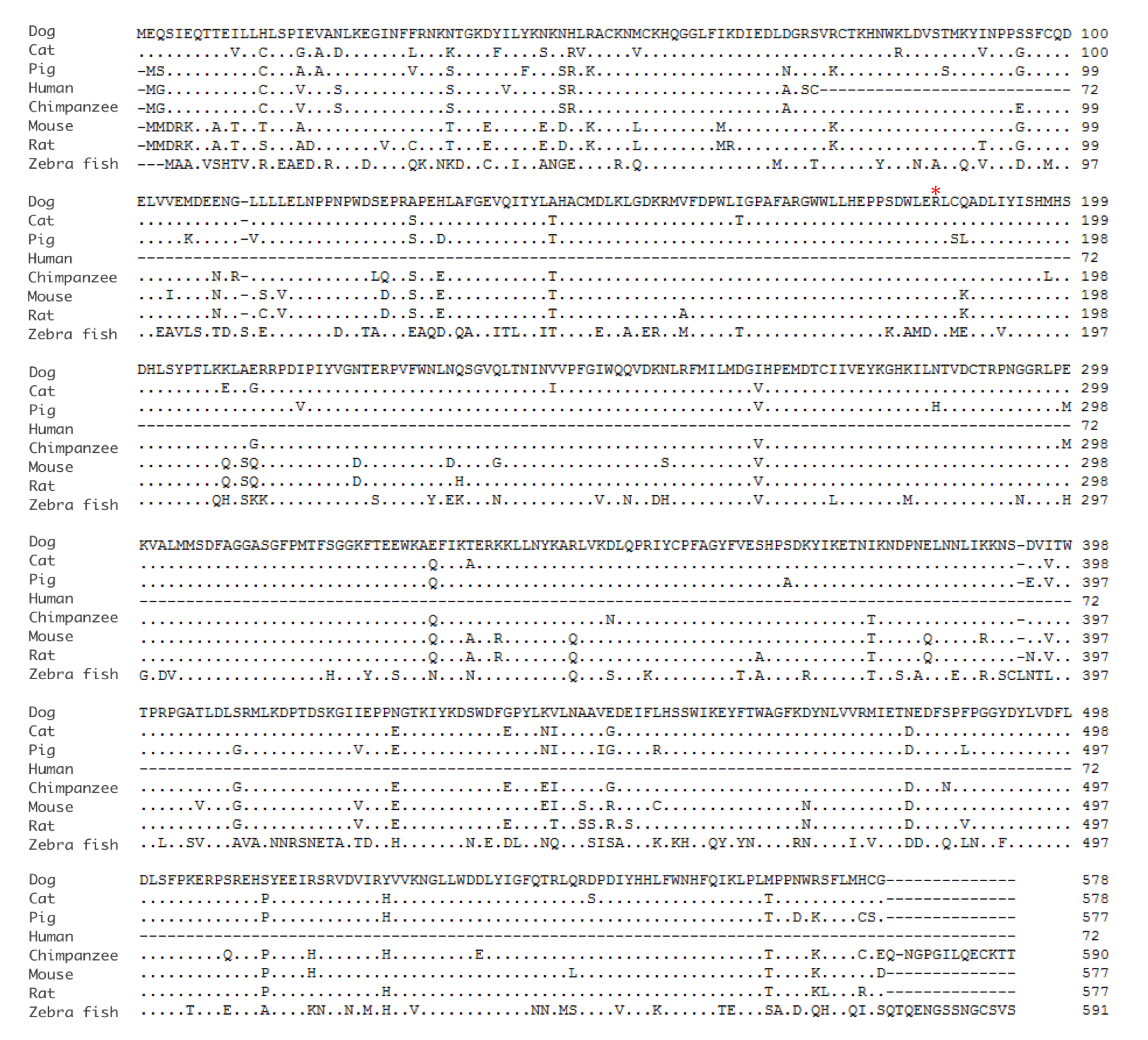


Dot (.)= same amino acid as dog CMAH,  dash (-)= deletion, * = position of amino acid residue at 185 corresponding to the SNP in dog

CMAH（p.Lys185Arg）
