# Supplementary material for: Molecular characterization of cytidine monophospho-N-acetylneuraminic acid hydroxylase (CMAH) associated with the erythrocyte antigens in dogs
Source: Canine Genet Epidemiol. 2019 Nov 7;6:9. doi: 10.1186/s40575-019-0076-1 (PMC6842231; doi:10.1186/s40575-019-0076-1)

Additional file 2 . Evolutionary tree generated by Unweighted Pair Group Method with Arithmetic Mean from the *CMAH* amino acid sequences using Genetyx-MAC.


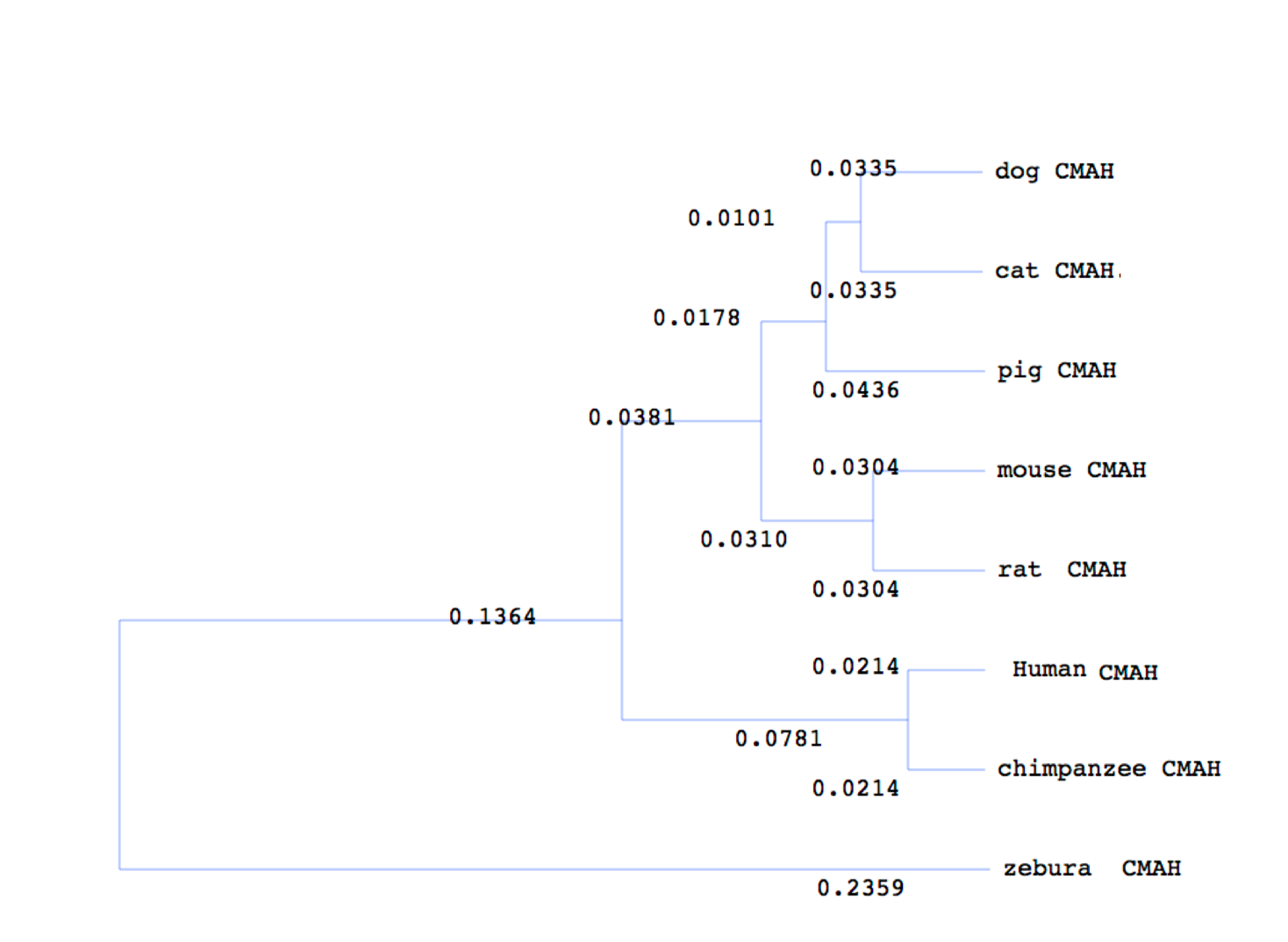


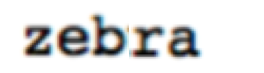

Supplement: Supplementary file 2 — Additional file 2 Evolutionary tree generated by Unweighted Pair Group Method with Arithmetic Mean from CMAH amino acid sequences using Genetyx-MAC. [file 40575_2019_76_MOESM2_ESM.docx]
