# Supplementary material for: Molecular characterization of cytidine monophospho-N-acetylneuraminic acid hydroxylase (CMAH) associated with the erythrocyte antigens in dogs
Source: Canine Genet Epidemiol. 2019 Nov 7;6:9. doi: 10.1186/s40575-019-0076-1 (PMC6842231; doi:10.1186/s40575-019-0076-1)
Supplement: Supplementary file 3 — Additional file 3. List of genotypes of 15 single nucleotide polymorphisms (4 exonic and 11 intronic) and an indel in 11 dogs, each from a different breed. Red character shows the nucleotide mutation revealed in this study. [file 40575_2019_76_MOESM3_ESM.docx]

Additional file 3. List of genotypes of 15 single nucleotide polymorphisms (4 exonic and 11 intronic) and an indel in 11 dogs, each from a different breed.

|  |  |  |  |  |  |  |  |  |  |
| --- | --- | --- | --- | --- | --- | --- | --- | --- | --- |
| **Breeds** | **LabID** | **exon 2 c.15T>C** | **intron2  c.216-18A>T** | **intron 2  c.216-17T>A** | **intron 4  c.418-77A>C** | **intron 4  c.418-76G>A** | **exon 5  c.554A>G** | **intron 6  c.756+71A>T** | **intron 9  c.1112+13G>A** |
| **Miniature dachshund** | **22717** | **TC** | **AT** | **TT** | **AC** | **GA** | **GG** | **AT** | **GG** |
| **Welsh corgi** | **22711** | **CC** | **AA** | **AA** | **AA** | **GG** | **GG** | **AT** | **GG** |
| **Labrador retriever** | **22733** | **CC** | **AA** | **AA** | **AA** | **GG** | **GG** | **AT** | **GG** |
| **Shetland sheep dog** | **22691** | **CC** | **AA** | **AA** | **AA** | **GG** | **GG** | **AT** | **GG** |
| **Beagle** | **22732** | **CC** | **AA** | **AA** | **AA** | **GG** | **GG** | **AT** | **GG** |
| **Yorkshire terrier** | **22775** | **TC** | **AT** | **AA** | **AA** | **GG** | **GG** | **AT** | **GG** |
| **Dobermann** | **22572** | **CC** | **AA** | **AA** | **AA** | **GG** | **GG** | **AT** | **AA** |
| **Whippet** | **21164** | **CC** | **AA** | **AA** | **AA** | **GG** | **GG** | **AT** | **GG** |
| **Weimaraner** | **21326** | **CC** | **AA** | **AA** | **CC** | **AA** | **GG** | **AT** | **AA** |
| **Papillon** | **22737** | **TC** | **AA** | **TA** | **AA** | **GG** | **GG** | **AT** | **GG** |
| **Shiba dog** | **TOTO** | **TC** | **AA** | **AA** | **AC** | **GA** | **AG** | **AA** | **GG** |
|  |  |  |  |  |  |  |  |  |  |
| **Breeds** | **LabID** | **intron 10 c.1253+52delT** | **intron 11 c.1428+51T>C** | **intron 12  c.1546-59G>A** | **intron 13  c.1662-92G>A** | **Intron 13  c.1662-12C>T** | **exon 14  c.1701G>A** | **exon 14  c.1713G>A** | **intron 14  c.1737+117A>G** |
| **Miniature dachshund** | **22717** | **T*** | **CC** | **GG** | **GG** | **CC** | **AA** | **GG** | **AA** |
| **Welsh corgi** | **22711** | **T*** | **CC** | **GG** | **AA** | **CC** | **GG** | **GG** | **GG** |
| **Labrador retriever** | **22733** | **T*** | **TT** | **GG** | **AA** | **CC** | **GG** | **GG** | **GG** |
| **Shetland sheep dog** | **22691** | **T*** | **TT** | **GG** | **GG** | **CC** | **GG** | **GG** | **AA** |
| **Beagle** | **22732** | **T*** | **TC** | **GG** | **GG** | **CC** | **GA** | **GG** | **AA** |
| **Yorkshire terrier** | **22775** | **T*** | **TC** | **GG** | **GA** | **CC** | **GA** | **GG** | **AG** |
| **Dobermann** | **22572** | **T*** | **CC** | **GG** | **GG** | **CC** | **AA** | **GG** | **AA** |
| **Whippet** | **21164** | **T*** | **CC** | **GG** | **AA** | **CC** | **GG** | **GG** | **GG** |
| **Weimaraner** | **21326** | **T*** | **TT** | **GG** | **GG** | **CC** | **GG** | **GG** | **AA** |
| **Papillon** | **22737** | **T*** | **CC** | **GG** | **GA** | **CC** | **GA** | **GG** | **AG** |
| **Shiba dog** | **TOTO** | **T*** | **TC** | **GA** | **GG** | **CT** | **GG** | **GA** | **AA** |

Red character shows the nucleotide mutation revealed in this study. These single nucleotide polymorphisms and an indel DNA polymorphisms of dog CMAH were shown in Figure 4.
