# Supplementary figures and images for: Molecular characterization of cytidine monophospho-N-acetylneuraminic acid hydroxylase (CMAH) associated with the erythrocyte antigens in dogs
Source: Canine Genet Epidemiol. 2019 Nov 7;6:9. doi: 10.1186/s40575-019-0076-1 (PMC6842231; doi:10.1186/s40575-019-0076-1)

Additional file 4 . RT-PCR analysis of the feline *CMAH* mRNA expression in different tissues of cat.


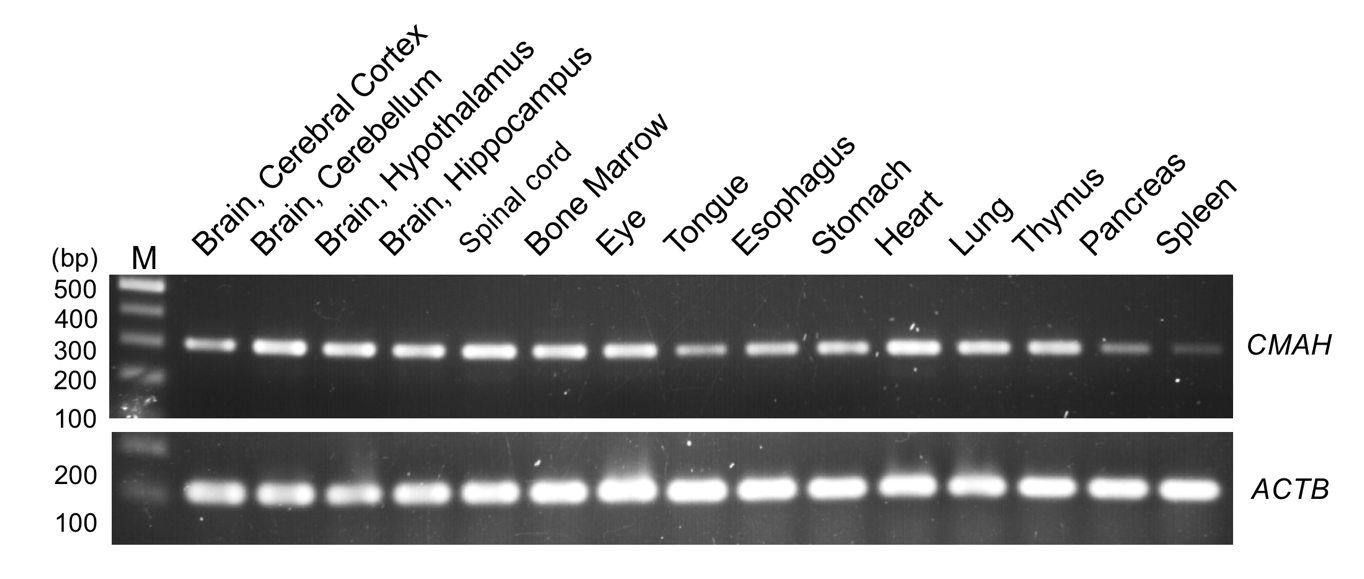


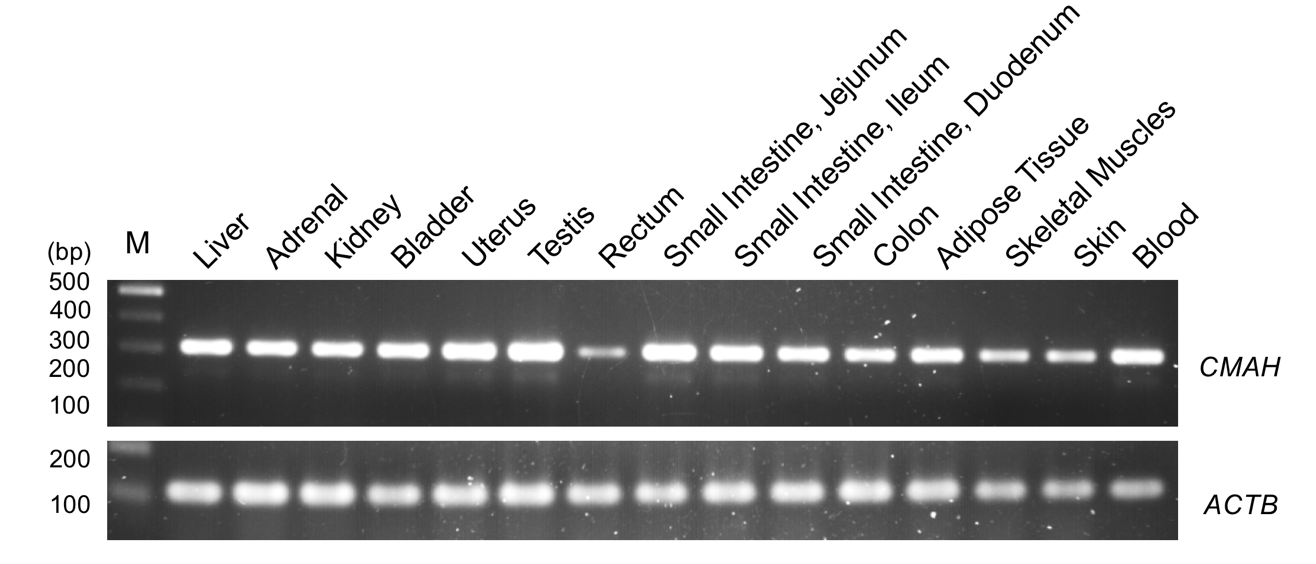

Supplement: Supplementary file 4 — Additional file 4 RT-PCR analysis of feline CMAH mRNA expression in different tissues of cat. [file 40575_2019_76_MOESM4_ESM.docx]
